# Relationship between Milk Protein Polymorphism and Selected Cows’ Reproductive Indices

**DOI:** 10.3390/ani13111729

**Published:** 2023-05-23

**Authors:** Ewa Czerniawska-Piątkowska, Barbara Cioch-Szklarz, Alicja Kowalczyk, Marcjanna Wrzecińska, Jerzy Wójcik, Władysław Kordan, Jose Pedro Araujo, Joaquim L. Cerqueira, Kamil Kossakowski, Przemysław Cwynar, Piotr Sablik

**Affiliations:** 1 Department of Ruminant Science, West Pomeranian University of Technology, Janickiego 29, 71-270 Szczecin, Poland; 2 Department of Environment Hygiene and Animal Welfare, Wrocław University of Environmental and Life Sciences, Chelmonskiego 38C, 51-630 Wroclaw, Poland; 3 Department of Animal Biochemistry and Biotechnology, University of Warmia and Mazury, 10-718 Olsztyn, Poland; 4 Mountain Research Centre (CIMO), Instituto Politécnico de Viana do Castelo, Rua D. Mendo Afonso, 147 Refóios do Lima, 4990-706 Ponte de Lima, Portugal

**Keywords:** Polish Holstein–Friesian cattle, milk protein polymorphism, kappa-casein, beta-lactoglobulin, reproductive indicators

## Abstract

**Simple Summary:**

Milk protein polymorphisms, especially κ-casein and β-lactoglobulin, are essential for the dairy and cheese industries. However, *BB CSN3* is a more favorable polymorphism, as it is responsible for a higher casein content. Statistically significant differences (*p* ≤ 0.05) in interpregnancy periods values were observed between cows with the *BB* kappa-casein genotype and cows with the *AA* genotype—the former exhibited the shortest interpregnancy periods. Additionally, cows with the *BB* genotype showed more favorable reproductive indices, i.e., the lowest age at first calving and the lowest insemination index. However, these values were not statistically significant. There was no significant influence of any of the analyzed kappa-casein and beta-lactoglobulin genotypes on the course of cows’ delivery.

**Abstract:**

This research sought to determine the effect of selected milk protein polymorphisms on the reproduction parameters of 598 black and white Polish Holstein–Friesian cattle. The analyzed genes were kappa-casein (*CSN3*) and beta-lactoglobulin (*BLG*). The following reproductive indexes were selected: the age at first calving, the interpregnancy period, the calving interval, and the insemination index. The influence of the identified genotypes on the course of parturition in cows was also analyzed. Source data were collected for each cow from the beginning of the herd life and reproduction to its culling from the herd or the end of its third lactation. Data on the age at first calving, the amount of semen portions for artificial insemination (insemination index), the interpregnancy period, and the calving interval for each cow were also collected. A contingency analysis was performed through contingency tables using a Pearson’s chi-squared test for each *CSN3* and *BLG* genotype. The results show that the *BB* genotype of the kappa-casein gene was associated with the most favorable values of reproductive indicators. In the case of the calving interval, the values were significantly more favorable than those of other genotypes (*p* ≤ 0.05). No effect of beta-lactoglobulin polymorphism on the analyzed reproductive indices was observed. On the other hand, in the case of the interpregnancy period, significant statistical differences were obtained between the *AA* and *BB* genotypes. The analyzed kappa-casein and beta-lactoglobulin genotypes did not significantly influence the course of parturition in cows. To conclude, the genotype polymorphism *BB CSN3* is the most favorable for the performance of the cows in the examined herd.

## 1. Introduction

Milk is considered an excellent food because it is rich in protein (2.9–3.5%), carbohydrates (5%), fat (3.8–5.5%), and minerals and vitamins, as well as free amino acids and conjugated linoleic acid (CLA), meeting the nutrient needs for developing offspring, for which milk proteins are considered the most valuable component [1,2]. This is important for production of fermented dairy drinks or cheese [3]. Milk proteins are divided into whey proteins, a soluble fraction constituting about 20% of all milk proteins (the most important are β-lactoglobulin and α-lactoglobulin). An insoluble casein fraction, comprising about 80% of proteins (αS1, αS2, β, and κ fractions), is the most crucial [4,5]. In milk, casein is the most critical component because, due to its high nutritional value, it plays a pivotal role in the rearing of offspring, being the primary source of calcium and phosphorus [5]. The share of β-lactoglobulin in the whey fraction of cow’s milk protein is about 50%, constituting 10% of all milk proteins.

Moreover, milk is most commonly associated with allergic reactions and is recognized by a specific IgE in milk-allergic patients [2]. These proteins are characterized by a high proportion of essential amino acids, determining their nutritional properties. They can emulsify lipids and bind water, improving the organoleptic properties of dairy products [2].

Milk protein polymorphisms, especially κ-casein and β-lactoglobulin, are economically significant [4]. In this regard, the kappa-casein (*CSN3*) and the beta-lactoglobulin (*BLG*) genes are notorious [6]. Kappa-casein and beta-lactoglobulin polymorphisms are associated with milk composition, cheese, fermented dairy drink production, and milk quality [6]. The polymorphism of the *CSN3* casein gene affects the course of the milk coagulation process and the resulting curd quality. A point mutation in exon IV of the *CSN3* gene determines two allelic variants, *A* and *B* [7]. β-lactoglobulin is the most diverse protein in a polymorphic form, but the most frequently identified alleles are *A* and *B* [7].

The gene encoding kappa-casein is essential from the perspective of milk processing technology. The polymorphism of *CSN3* influences the course of technological processes in cheese making. For example, the authors of many publications [7,8,9,10] indicate a shorter coagulation time under the influence of rennet and a greater firmness of the resulting milk curd from cows with the *BB CSN3* genotype. Similarly, the results of numerous studies have shown that the milk of cows with the *BB* and *AB CSN3* genotypes, compared to the milk of cows with the *AA CSN3* genotype, is characterized by a 10–30% shorter coagulation time, a 20–100% greater firmness of the formed curd, a 5–8% higher yield of parmesan and cheddar cheese, and a 2–4% higher total nitrogen conversion in cheddar, camembert, and gouda cheeses. These results allowed for the recognition of *CSN3* as a gene with a beneficial and pleiotropic solid effect [11,12].

Research on dairy cattle in many countries has shown that the fat content is low and systematically declines where there is the prevalence of the beneficial *CSN3* allele *B* in terms of total protein, including casein [7,9,10,11]. Dairy cattle insemination stations in Poland, Europe, and the USA place milk protein genotypes in bull catalogs, including kappa-casein for each bull, presented as a genetic marker [13,14].

While many studies have investigated the effect of genetic polymorphisms of milk proteins on milk production traits, only a few have investigated their relationship with the reproductive performance of cattle [15]. For example, a study conducted by Jairam and Nair [16] reported a lower first calving age for heifers with the *AB CSN3* and *AB BLG* genotypes. In contrast, other researchers found no significant associations between kappa-casein and beta-lactoglobulin genotypes and reproductive performance [17,18]. Another study showed that cows with the *AB BLG* genotype had a more extended gestation period, while the age of first calving was lower in animals with the *AB BLG* genotype [19]. The study showed no significant effect of the *CSN3* genotype on any analyzed reproductive traits. The literature lacks data on the impact of the polymorphism of the referred genes on reproduction indices.

This article aims to determine the effect of selected milk protein (kappa-casein and beta-lactoglobulin) polymorphisms on the reproduction parameters of 598 black and white Polish Holstein–Friesian cattle.

## 2. Materials and Methods

This research included 598 Polish Holstein–Friesian (PHF) black and white cattle assessed by the Polish Federation of Cattle Breeders and Milk Producers. Cows came from one of the dairy farms in Wielkopolskie voivodeship and were kept in a free-stall barn. The animals were fed using the TMR (Total Mixed Ration) system. Source data were collected for each cow from the beginning of the herd life and reproduction to its culling from the herd or to the end of its third lactation. The average yield of the cow population of this breed was 8298 kg of milk, with a fat content of 4.03% and a protein content of 3.39%.

The experiment was performed as part of routine clinical activities in the herd based on monitoring the health of the females. Each animal’s blood was collected from the jugular vein in a sterile tube with ethylenediamine tetraacetic acid (EDTA). First, a veterinarian collected the biological material. After collecting the blood, samples were gently mixed and transported to the laboratory. The genetic material was isolated, which was achieved using a MasterPure™ Genomic DNA Purification Kit for Blood Version II by Epicentre Technologies. PCR was performed to amplify the DNA, and PCR-RFLP was used for genotype analyses. Then, the amplification products were digested with an appropriate restriction enzyme (*Hind*III for the *CSN3* gene and *Hae*II for the *BLG* gene), and the analysis results were imaged on an agarose gel. The study included a polymorphism analysis of two selected genes: *CSN3* and *BLG*. The characteristics of the selected polymorphic sites are presented in Table 1.

Source data were collected for each cow from the start of the cows’ reproductive performance until the day of culling or until the end of the third lactation. On-farm methods for detecting estrus included using devices to monitor changes in activity and the daily observation of animal behavior. Motion sensors were mounted on the hind limb of the cow (the so-called pedometers), i.e., sensors placed on the stems of cows. The population of experimental cows was divided into two groups taking into account the genotypes of the studied *loci*. For each animal, information was collected on the age at first calving, the amount of semen portions for artificial insemination (insemination index), the interpregnancy period (the period from calving to successful insemination/mating), and the calving interval. The course of parturition was also analyzed (ease of calving). The following calving scores were determined: 1—self-dependent; 2—easy; 3—difficult with more strength than normal; 4—hard (surgical intervention, injury of the cow or calf, or embryotomy); and 5—abortion [20]. A contingency analysis was performed through contingency tables, using a Pearson’s chi-squared test separately for each analyzed *CSN3* and *BLG* genotype. Statistical calculations were performed using the SAS/STAT [21] program.

## 3. Results

### 3.1. Relationship between Kappa-Casein Polymorphism and Reproductive Indicators

The relationship between the polymorphism of the kappa-casein (*CSN3*) gene and the reproductive traits is presented in Table 2.

The values of the selected reproductive indicators for the examined dairy herd with different genetic variants of *CSN3* are presented in Table 2. In the analyzed herd, the best reproduction rates were obtained for cows with the *BB* genotype, which registered the lowest age at the first calving (712.21 days), the lowest insemination index (3.04), and the shortest interpregnancy periods (142.50 days) and calving interval (411.57 days). Moreover, the value of the calving interval for cows with the *BB* genotype was significantly different (*p* ≤ 0.05) from the values of other genotypes, while the value of the interpregnancy period was significantly different (*p* ≤ 0.05) from the value of the *AA* genotype (Table 2).

The shortest calving interval was observed in homozygous *BB* females (411.57 days), followed by heterozygous females (422.79 days), whereas the longest calving interval was observed in cows with the *AA* genotype (433.77 days) (Table 2).

In the examined herd of cows, those with the *BB* genotype had a significantly (*p* ≤ 0.05) shorter interpregnancy period (142.5 days) than the ones with an *AA* homozygous genotype (149.94 days) (Table 2).

The insemination index in the examined herd ranged from 3.04 for *BB* homozygotes to 3.32 for *AA* heterozygotes (Table 2).

The course of parturition, including the kappa-casein genotype, is presented in Table 3.

Table 3 presents the evaluation of the course of the parturition of cows in three consecutive calvings, including the kappa-casein genotype. No significant influence of the analyzed genotypes on the course of parturition was found as a result of the statistical analysis.

### 3.2. Relationship between Beta-Lactoglobulin Polymorphism and Reproductive Indicators

Table 4 shows the relationship of the selected reproductive factors with the beta-lactoglobulin genotypes. 

Table 4 presents the values of the selected reproduction indexes of the analyzed dairy herd, including the different genetic variants of beta-lactoglobulin. During the statistical analysis, no statistically significant influence of the examined polymorphism on cow fertility was reported.

The course of parturition, including the beta-lactoglobulin genotype, is presented in Table 5.

Table 5 shows an evaluation of the course of parturition in the three consecutive calves, including the beta-lactoglobulin genotype. During the statistical analysis, no significant effect of the examined genotypes on the course of parturition was observed.

## 4. Discussion

### 4.1. Relationship between Kappa-Casein Polymorphism and Reproductive Indicators

A correctly selected age at the first calving increases milk yield in the first lactation and improves the effectiveness of pregnancy [22]. According to Sawa et al. [23], cows calving at 26 months of age are characterized by the highest milk yield in the first lactation compared to cows calving over 30 months of age. According to Eastham et al. [22], the age at the first calving affects the first lactation, but its effect on subsequent lactations is less significant. Sitkowska et al. [24] demonstrated that cows that calved between 26 and 30 months of age exhibited the highest milk, fat, and protein yield in their first lactation. However, for the second and third lactations, cows calving between 24 and 26 months of age demonstrated the best milk, fat, and protein yield performance. Some authors argue that the age at the first calving correlates with the milk yield and the length of the cows’ productive life in the herd [22,25]. The earliest calving cows (up to 25 months of age) have the longest length of productive life. Czerniawska-Piątkowska et al. [26] report that the PHF black and white variety cow calve for the first time at the age of 822 days, i.e., about 27 months. In their research, cows with the *BB CSN3* genotype demonstrated a lower age at the first calving (712.21 days or 24 months).

In their study, Asim et al. [27] showed that the polymorphisms of the *CSN3* gene are associated (*p* < 0.05) with the calving interval in Sahiwal cattle. In other studies, the influence of the kappa-casein genotype on the calving interval was not shown. Still, a longer calving interval was detected for the *AB* genotype compared to the *AA* genotype of this gene [15]. Research conducted by Miciński et al. [28] showed that heterozygous cows were also characterized by significantly shorter calving intervals than homozygous *AA* cows (464 and 413 days, respectively). These results are also similar to those obtained in our research. The calving interval is one of the most essential reproduction indexes. It has been shown that the lengthening of the calving interval is accompanied by an increase in milk yield [29]. However, an excessively long calving interval may lead to economic losses [30]. A calving interval of about 360–400 days is considered optimal; an extension of the calving interval indicates a reproductive disorder in cows [30].

Similar differences between the genotypes *BB* and *AA* regarding the interpregnancy period were obtained by Miciński et al. [28], who reported a significantly (*p* ≤ 0.05) shorter calving interval for heterozygotes (141 days) in comparison with *AA* homozygotes (182 days). Cows with interpregnancy intervals between 80 and 120 days are commonly associated with higher production rates. According to Miciński et al. [31], the optimal length of the interpregnancy period should be 110–130 days. Cows with an average interpregnancy period length ranging from 121 to 160 days were characterized by the highest lifetime milk, fat, and protein yields [32,33]. Studies have shown that an increase in milk yield from less than 7000 kg to over 8000 kg was associated with an extension of the interpregnancy period from 154.26 to 171.48 days [31].

The values of the insemination index presented in Table 2 significantly exceeded the adopted norms (Table 2). Miciński et al. [28] reported that cows with the *AB* genotype (2.04) had a significantly (*p* ≤ 0.05) lower insemination index compared to cows with the *AA* genotype (3.15). The authors put forward a hypothesis that negative values of reproductive parameters in the herd may indicate fertility problems.

It is also reported that cows producing a milk yield in the 8000–8500 kg range obtained a high unfavorable insemination index value of 2.5 [32,34,35]. The insemination index is considered to be negative if it is over 2.5 [35]. Such a high insemination index is related to the increased milk yield of cows because insemination coincides with the peak milk production and potential energy, protein, and mineral deficits. These deficiencies affect the hypothalamus and pituitary gland, the quality of oocytes, and the functions of the corpus luteum, causing an extension to the interpregnancy period and the ineffectiveness of insemination [36].

The data presented in Table 3 show that easy calving accounted for over 90% of all parturitions in the first and the second calvings and almost 89% in the third calving. Such a high proportion of easy calving should be assessed positively; usually, in dairy cows, the percentage of easy parturition is around 90% [37]. Self-dependent calving in each subsequent parturition accounted for only a few percentage points—the lowest occurred in the first calving (3.19%) and the highest in the third calving (6.34%). The low percentage of self-dependent calving results from special control measures used for cows about to calve rather than the necessity to provide assistance to calving cows. Difficult parturition in the population was very rare (0.84–3.07%). The percentage of difficult calving in PHF cattle varies from 3 to 13%; in turn, the mortality in calves ranges from 2.5 to 10% [37,38]. Pogorzelska and Nogalski [39] conducted a population study on the incidence of difficult calving in 2010 in Poland. At the time, difficult calving in cows occurred with a frequency of 3.86%, while perinatal calf mortality was 5.62%. The percentage of parturitions requiring veterinary assistance was between 5 and 7% [37].

In all three consecutive calves, the easiest calving was reported for cows with the *AA* genotype, followed by the *AB* and then the *BB* genotype. In addition, for all miscarriages that occurred in cows with the *AA* and *AB* genotypes, no abortions were noted for the *BB* genotype.

### 4.2. Relationship between Beta-Lactoglobulin Polymorphism and Reproductive Indicators

Homozygous *AA* cows are characterized by the lowest insemination index and the shortest interpregnancy period (3.12 and 145.28 days, respectively). Cows with the *BB* genotype calved the earliest (784.8 days of life), and the shortest calving interval was registered in *AB* heterozygotes (422.04 days). Fertility rates in the analyzed herd, including polymorphism of *BLG*, also exceeded the favorable average, except for the age at the first calving, which was reasonable. There were no statistically significant differences.

The data presented in Table 5 show that cows with the *AB* genotype were characterized by an abundance of easy calving, with more than 45% of such cases in each parturition. In all three consecutive calvings, easy calving was most often reported for cows with the *AB* and *AA* genotypes and the least reported for the *BB* genotype. Miscarriages were observed in every genotype.

## 5. Conclusions

In this research, cows with the *BB* kappa-casein genotype displayed more favorable reproductive indicators compared to other genotypes, including the shortest calving intervals. Moreover, this genotype was associated with the statistically shortest interpregnancy period compared to the *AA* genotype. No statistically significant differences were observed in terms of the influence of beta-lactoglobulin polymorphism on fertility rates. Furthermore, none of the analyzed genotypes of kappa-casein and beta-lactoglobulin significantly impacted the course of parturition in cows. Nevertheless, the current literature needs more data on the effect of the polymorphism of the studied genes on reproduction indices, highlighting the need for further research in this area.

## Figures and Tables

**Table 1:** Characteristics of the analyzed polymorphic sites in the *CSN3* and *BLG* genes.

Gene	Chromosome	Mutation Site	Type of Mutation
*CSN3*	6	exon 4	*A*→*C*
*BLG*	11	exon 4	*C→T*

**Table 2:** Values of selected cow reproduction indices, including *CSN3* genotypes.

Traits	*CSN3*								
	*AA*			*AB*			*BB*		
	*n*	x	SD	*n*	x	SD	*n*	x	SD
Age at first calving (days)	354	717.82	76.72	206	716.57	74.91	18	712.21	63.55
Insemination index	876	3.32	2.37	510	3.26	2.33	45	3.04	2.5
Calving interval (days)	349	433.77 ^a^	87.45	628	422.79 ^b^	92.03	330	411.57 ^c^	108.07
Interpregnancy period (days)	819	149.94 ^a^	64.73	466	145.26	62.29	44	142.5 ^b^	58.35

^a, b, c^ statistically significant differences (significance level *p* ≤ 0.05); SD—standard deviation.

**Table 3:** The course of parturition in the examined population of cows, including kappa-casein genotypes.

*CSN3*		Parturition Code					
		1	2	3	4	5	Σ
First calving							
*AA*	*n*	15	342	2	-	4	363
	%	2.52	57.48	0.34	-	0.67	61.01
*AB*	*n*	4	201	3	-	6	214
	%	0.67	33.78	0.50	-	1.01	35.97
*BB*	*n*	-	18	-	-	-	18
	%	-	3.03	-	-	-	3.03
Σ	*n*	19	561	5	-	10	595
	%	3.19	94.29	0.84	-	1.68	100
Pearson’s chi-squared test						*p* = 0.34	
Second calving							
*AA*	*n*	20	324	4	1	7	356
	%	3.44	55.77	0.69	0.17	1.20	61.27
*AB*	*n*	11	192	2	-	3	208
	%	1.89	33.05	0.34	-	0.52	35.80
*BB*	*n*	2	15	-	-	-	17
	%	0.34	2.58	-	-	-	2.93
Σ	*n*	33	531	6	1	10	581
	%	5.68	91.39	1.03	0.17	1.72	100
Pearson’s chi-squared test						*p* = 0.96	
Third calving							
*AA*	*n*	21	264	10	-	7	302
	%	4.29	53.99	2.04	-	1.43	61.76
*AB*	*n*	10	153	5	-	3	171
	%	2.04	31.29	1.02	-	0.61	34.97
*BB*	*n*	-	16	-	-	-	16
	%	-	3.27	-	-	-	3.27
Σ	*n*	31	433	15	-	10	489
	%	6.34	88.55	3.07	-	2.04	100
Pearson’s chi-squared test						*p* = 0.85	

%—participation in genotype; Parturition code: 1—self-dependent, 2—easy, 3—difficult with more strength than normally, 4—hard (surgical intervention, injury to the cow or calf, or embryotomy), 5—abortion.

**Table 4:** Values of the selected reproduction indices, including beta-lactoglobulin genotypes (*BLG*).

Traits	*BLG*								
	*AA*			*AB*			*BB*		
	*n*	x	SD	*n*	x	SD	*n*	x	SD
Age at first calving (days)	149	799.7	72.2	301	794.6	77.9	127	784.8	85.7
Insemination index	373	3.12	2.24	740	3.32	2.43	318	3.42	2.33
Interpregnancy period (days)	351	145.28	60.71	682	148.4	64.02	296	150.54	66.4
Calving interval (days)	267	430.87	100.98	519	422.04	87.64	221	430.6	86.19

SD—standard deviation.

**Table 5:** The course of parturition in the examined population of cows, including beta-lactoglobulin genotypes.

*BLG*		Parturition Code					
		1	2	3	4	5	Σ
First calving							
*AA*	*n*	5	147	1	-	1	154
	%	0.85	24.71	0.17	-	0.17	25.88
*AB*	*n*	11	289	3	-	6	309
	%	1.85	48.57	0.50	-	1.01	51.93
*BB*	*n*	3	125	1	-	3	132
	%	0.50	21.01	0.17	-	0.50	22.18
Σ	*n*	19	561	5	-	10	595
	%	3.19	94.29	0.84	-	1.68	100
Pearson’s chi-squared test						*p* = 0.92	
Second calving							
*AA*	*n*	9	142	1	-	3	155
	%	1.55	24.44	0.17	-	0.52	26.68
*AB*	*n*	17	275	2	1	4	299
	%	2.93	47.33	0.34	0.17	0.69	51.46
*BB*	*n*	7	114	3	-	3	127
	%	1.20	19.62	0.52	-	0.52	21.85
Σ	*n*	33	531	6	1	10	581
	%	5.68	91.39	1.03	0.17	1.72	100
Pearson’s chi-squared test						*p* = 0.82	
Third calving							
*AA*	*n*	10	112	4	-	4	130
	%	2.04	22.90	0.82	-	0.82	26.58
*AB*	*n*	15	223	6	-	4	248
	%	3.07	45.60	1.23	-	0.82	50.72
*BB*	*n*	6	98	5	-	2	111
	%	1.23	20.04	1.02	-	0.41	22.70
Σ	*n*	31	433	15	-	10	489
	%	6.34	88.55	3.07	-	2.04	100
Pearson’s chi-squared test						*p* = 0.84	

%—participation in genotype; Parturition code: 1—self-dependent, 2—easy, 3—difficult with more strength than normally, 4—hard (surgical intervention, injury to the cow or calf, or embryotomy), 5—abortion.

## Data Availability

Not applicable.
